# The effect of different weight loss strategies to treat non-alcoholic fatty liver disease focusing on fibroblast growth factor 21

**DOI:** 10.3389/fnut.2022.935805

**Published:** 2022-08-10

**Authors:** Nicole Power Guerra, Katharina Leyens, Luisa Müller, David Brauer, Deborah Janowitz, Samin Schlick, Kristin Pilz, Hans J. Grabe, Brigitte Vollmar, Angela Kuhla

**Affiliations:** ^1^Institute for Experimental Surgery, Rostock University Medical Center, Rostock, Germany; ^2^Institute of Anatomy, Rostock University Medical Center, Rostock, Germany; ^3^Department of Psychosomatic Medicine, Rostock University Medical Center, Rostock, Germany; ^4^Department of Systems Biology and Bioinformatics, University of Rostock, Rostock, Germany; ^5^Department of Psychiatry, University Medicine Greifswald, Greifswald, Germany; ^6^Clinic for Psychiatry and Psychotherapy, HELIOS Hanseklinikum Stralsund, Stralsund, Germany

**Keywords:** non-alcoholic fatty liver disease, high-fat diet, dietary change, treadmill exercise, time-restricted feeding, FGF21, TNFα, β-klotho

## Abstract

**Objective:**

Obesity, often associated with non-alcoholic fatty liver disease (NAFLD), is characterized by an imbalance between energy expenditure and food intake, which is also reflected by desensitization of fibroblast growth factor 21 (FGF21). FGF21 is strongly influenced, among others, by TNFα, which is known to be upregulated in obesity-induced inflammation. Successful long-term treatments of NAFLD might be dietary modification, exercise, or fasting.

**Materials and methods:**

Whether succeeded NAFLD recovery is linked with improved FGF21 sensitivity and finally reverted FGF21 resistance was the focus of the present study. For this purpose, mice received a high-fat diet (HFD) for 6 months to establish obesity. Afterward, the mice were subjected to three different weight loss interventions, namely, dietary change to low-fat diet (LFD), treadmill training, and/or time-restricted feeding for additional 6 months, whereas one group remained on HFD.

**Results:**

In addition to the expected decrease in NAFLD activity with dietary change, this was also observed in the HFD group with additional time-restricted feeding. There was also an associated decrease in hepatic TNFα and FGF21 expression and an increase in ß-klotho expression, demonstrated mainly by using principal component analysis. Pearson correlation analysis shows that independent of any intervention, TNFα expression decreased with improved NAFLD recovery. This was accompanied with higher FGF21 sensitivity, as expressed by an increase in β-klotho and FGFR1c expression and concomitantly decreased FGF21 levels.

**Conclusion:**

In summary, we conclude that successful NAFLD therapy is associated with a reversion of the TNFα-triggered FGF21-resistant state or desensitization.

## Introduction

Overall, 25% of people worldwide suffer from overweight or obesity, which has reached pandemic proportions. As early as 1989, Kaplan described the “deadly quartet” of abdominal obesity, hypertension, hyperglycemia, and hypertriglyceridemia (1), which is referred to as metabolic syndrome (2, 3). The metabolic syndrome is associated with non-alcoholic fatty liver disease (NAFLD), which is considered as hepatic manifestation of this disease (4–6). One potential reason for the prevalence of NAFLD and many other comorbidities and sequelae of obesity is the persistence of a systemic low-grade inflammation (LGI) (7, 8). In this context, white adipose tissue is capable of expressing both metabolic and immunological mediators (9), which act locally but may also have systemic effects affecting other organs, such as the liver. This is reflected by hepatic and also systemic upregulation of pro-inflammatory cytokines, such as interleukin (IL)-1β, IL-6, and tumor necrosis factor alpha (TNFα) (10–12). All these mediators are well coordinated and reciprocally regulated in signaling cascades. An imbalance of these mediators is likely responsible for an LGI-mediated interaction between obesity and NAFLD (13, 14).

In order to better understand the causality of obesity-related inflammatory processes, it is necessary to consider individual hormones, such as leptin, ghrelin, or orexin. They are involved in the regulation of food intake. A dysregulation of these hormones, also triggered by obesity-induced LGI, can further aggravate obesity. In addition, fibroblast growth factor 21 (FGF21), a hormone in addition to fatty acid oxidation, lipolysis, and increased energy dissipation, is also involved in the regulation of food uptake. FGF21 acts *via* its receptor complex of FGF21 receptor (FGFR)1c and β-klotho in an endocrine or paracrine manner (15–17).

Remarkably, on the one hand, FGF21 expression is increased in the liver during fasting states and caloric restriction (18, 19); on the other hand, exceptionally high circulating plasma FGF21 concentrations occur in obese humans and mice (20, 21). Termed the “FGF21 paradox” by Fisher et al. (21), this phenomenon describes, similar to leptin resistance (22), an FGF21-resistant state (21), although more recently, the term FGF21 desensitization has been used in this context (23). Thereby, increased FGF21 concentrations in obesity were associated with a concomitant reduction in the FGF21 receptor complex (21). A link between FGF21 and inflammation was demonstrated by Diaz-Delfin and coworkers in mouse adipocytes, where the application of TNFα inhibited β-klotho expression (24). Furthermore, a long-term study on the adipose tissue of obese mice demonstrated that expression of FGFR1c and β-klotho was markedly decreased and associated with a limited effect of exogenously applied FGF21, implying a decrease in FGF21 sensitivity.

To overcome the vicious cycle of obesity and hence obesity-induced LGI, intervention approaches, such as physical activity, dietary changes, or fasting are appropriate and commonly used methods (25–27). Therefore, the purpose of this study was to investigate to which extent obesity-associated NAFLD and accompanying inflammatory processes are reversible by treadmill training, dietary change, and/or time-restricted feeding, and whether this is associated with enhanced hepatic FGF21 sensitivity. Using principal component and correlation analyses, we tested the hypothesis that recovery from NAFLD is associated with a reversion of FGF21 resistance or desensitization.

## Materials and methods

### Animals

**FIGURE 1:**
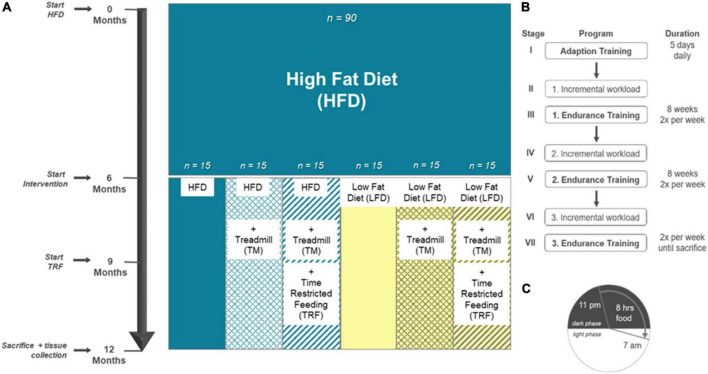
Experimental design adapted to Power Guerra et al. (28). **(A)** Female C57BL/6J mice (*n* = 90) were fed for 6 months with high-fat diet (HFD) to establish the model of diet-induced obesity. Thereafter, mice were divided into six groups. The first group remained on the HFD (*n* = 90). Groups 2–6 underwent an intervention. Second group: HFD plus treadmill exercise (TM; HFD/HFD + TM, *n* = 15); third group: HFD plus treadmill exercise and time-restricted feeding (TRF; HFD/HFD + TM + TRF, *n* = 15); fourth group: dietary change to a low-fat diet (LFD; HFD/LFD, *n* = 15); fifth group: dietary change plus treadmill exercise (HFD/LFD + TM, *n* = 15), and sixth group: dietary change, treadmill training, and time-restricted feeding (HFD/LFD + TM + TRF, *n* = 15). When dietary change was completed, treadmill training started. After 3 months of endurance exercise, time-restricted feeding was introduced. At the end, the mice were killed, and blood and liver tissue were collected. **(B)** Treadmill protocol consists of seven stages with three endurance sections. Prior to endurance training, an incremental workload test was performed to adjust the maximum velocity of the run. **(C)** Mice in the TRF group were restricted food from 7 a.m. to 11 p.m. (16 h). Food supply was provided in the nocturnal active phase for 8 h.

At the beginning, 90 4-week-old female mice (C57BL/6J) were purchased from Charles River (Sulzfeld, Germany). In compliance with our previous and ongoing investigations, female mice were used for comparability between different studies (28). All animal experimental work was carried out with permission of the local Animal Research Committee [Landesamt für Landwirtschaft, Lebensmittelsicherheit und Fischerei (LALLF) of the state Mecklenburg-Western Pomerania (LALLF M-V/TSD/7221.3-2-001/18, approved on March 1, 2018)] and by following the ARRIVE guidelines. All animals received human care according to the EU Directive 2010/63/EU. The mice were divided in a blinded manner into groups of five mice and were kept in standard cages. The room temperature was controlled (21 ± 3°C), and a 12-/12-h day/night cycle (lights on from 6:00 a.m. to 6:00 p.m.) was applied. All mice were handled equally for the first 6 months while establishing the model of diet-induced obesity. Therefore, all 90 mice received a high-fat diet (HFD; D12492; Research Diets, New Brunswick, NJ, United States) for 6 months *ad libitum*. According to randomization, the cages were divided into six groups. In the following 6 months, interventions were carried out as previously published by our group (28). The first group (*n* = 15) remained on an HFD, referred to as “HFD/HFD”. In the second group, named “HFD/HFD + TM”, treadmill (TM) exercise (TM 303401; TSE Systems Inc., Chesterfield, MO, United States) (*n* = 15) was added. The third group (*n* = 15) was trained on treadmills, and additionally, time-restricted food (TRF) intake was introduced after 3 months of intervention, designated as group “HFD/HFD + TM + TRF”. The HFD was changed to a low-fat diet (LFD; D12450J; Research Diets, New Brunswick, NJ, United States) for (*n* = 15), building the fourth group “HFD/LFD”. The fifth group (*n* = 15), called “HFD/LFD + TM”, also changed diet to an LFD in addition to treadmill exercise for time of interventions. The last group (*n* = 15) underwent all given interventions, named “HFD/LFD + TM + TRF”. Figure 1A illustrates the experimental design. Body weight was measured weekly and just before killing of the mice.

### Interventions

#### Dietary change to low-fat diet

For the first intervention, 45 mice received an LFD, containing 10% fat, 20% protein, and 70% carbohydrates, matching the HFD in structure of lard and protein composition. Contrary to this, the HFD consists of 60% fat, 20% protein, and 20% carbohydrates.

#### Treadmill exercise

In addition to dietary change, TM exercise was established for *n* = 60 mice as the second intervention parameter. The exact TM protocol was previously described by our group (28). In brief, TM exercise was performed twice a week, running through a program, as shown in Figure 1B.

#### Time-restricted food

To treat obesity and metabolic disorders, time limited restriction of food is described as a beneficial method (29, 30), which was the third intervention parameter, namely, TRF. After the third phase of TM exercise (Figure 1C), TRF was introduced to *n* = 30 mice using the same protocol, as previously described by our group (28). For the last 3 months, TRF was maintained. Food regulation was performed by using an autofeeder (EHEIM, Deizisau, Germany) with an enlarged opening. The food drop was controlled at 11 p.m. *via* a webcam with infrared light. The mice were transferred back to fresh cages at 7 a.m. with water supply and no enrichments.

### *In vitro* experiment

The human hepatoma cell line HepG2 was used for *in vitro* experiments and was cultured as reported by Guy et al. (31). The cells were seeded in six-well plates. After reaching 95% confluence, cells were incubated with 20 μg/mL human TNFα (hTNFα; Sigma Aldrich, Taufkirchen, Germany) or Aqua Dest (B. Braun Melsungen AG, Melsungen, Germany) for 24 h. Afterward, the cells were harvested for analysis of β-klotho protein expression.

### Sampling and assays

Under anesthesia (5 vol.% isoflurane; Baxter, Unterschleißheim, Germany), the mice were exsanguinated *via* retrobulbar puncture. Blood was collected and prepared according to Power et al. (31). Thereafter, a laparotomy was performed. The visceral and subcutaneous flanked fat deposits and liver tissue were harvested and weighted. Subsequently, the left lateral liver lobe was fixed in 4% paraformaldehyde (PFA, sc281692 Santa Cruz Biotechnology Inc., Dallas, TX, United States) for 5 days and embedded in paraffin (Carl Roth, Karlsruhe, Germany). The left medial lobe was embedded in Tissue-Tek^®^ (Sakura Finetek Germany GmbH, Umkirch, Germany), snap-frozen in liquid nitrogen with the remaining tissue, and stored at −20°C. For the assessment of liver damage, plasma alanine aminotransferase (ALT), aspartate aminotransferase (AST), and albumin activities were spectrophotometrically determined (Cobas c111; Roche Diagnostics, Mannheim, Germany) using commercially available reaction kits (Roche Diagnostics, Mannheim, Germany). Measurements of the LDL/VLDL fraction, triglycerides, leptin, insulin, and FGF21 in plasma were performed using the LDL/VLDL cholesterol, leptin, insulin, and FGF21 assay kits according to the manufacturers’ instructions (LDL/VLDL: Abcam, Cambridge, United Kingdom; leptin, insulin, FGF21: R&D System, Minneapolis, MN, United States; triglycerides: Cayman Chemical Company, Ann Arbor, MI, United States).

### Histology, immunohistochemistry, and image analysis

Hematoxylin and eosin (H&E) staining (Merck, Darmstadt, Germany) was performed using standard protocols on 4 μm thin tissue sections. Images were recorded on a Carl Zeiss Axioskop 40 microscope (Carl Zeiss AG, Oberkochen, Germany) with a Zeiss AxioCamMRc5 camera (Carl Zeiss AG, Oberkochen, Germany) and corresponding Zeiss ZEN2 lite software (Carl Zeiss AG, Oberkochen, Germany).

From the H&E-stained specimen, the NAFLD Activity Score (NAS) was assessed in a blinded manner to characterize diet-induced liver damage. Following the description by Kleiner et al. (32) and our previous work (33), the parameters steatosis (scores 0–3), hepatocellular ballooning (scores 0–2), and lobular inflammation (scores 0–3) were used to calculate the NAS (total scores 0–8). Steatosis was assessed at 50× magnification and ballooning at 100× magnification. Inflammation was assessed by counting inflammatory foci from 10 representative low-power fields (LPF) (200× magnification), characterized as a grouping of at least five inflammatory cells in the tissue that are not arranged in a row (34). For Oil Red O staining of lipids, the frozen liver tissue was cut in 8 μm thick sections, air-dried, and fixed in paraformaldehyde. The staining was performed using Oil Red O (Sigma-Aldrich Corp., St. Louis, MO, United States) and counterstained with hematoxylin. In total, 10 images at 400× magnification were taken per sample. Quantitative analysis of the red-stained area was conducted using ImageJ (v 1.52, Wayne Rasband, National Institutes of Health, United States) (protocol provided in the supplements as ImageJ Code S1) analyzing the percentage of red pixels per image.

To substantiate inflammatory processes in the liver, naphthol-AS-C-chloracetate esterase (CAE) staining (Sigma Aldrich Corp., St. Louis, MO, United States) was used for characterizing granulocytes. After fixing in paraformaldehyde and embedding, sections were stained with CAE and counterstained with hematoxylin. The ratio of CAE-positive cells (CAE+) and the total number of hepatocytes in 10 consecutive high power fields (HPF) at 400× magnification were used to quantify granulocytes in a blinded manner. Macrophages were immunohistochemically stained for the indication of cellular hepatic LGI. Therefore, overnight incubation (4°C) with a rat anti-mouse-F4/80 (MCA497; Bio-Rad, Hercules, CA, United States) was followed by 1 h incubation at room temperature with the second antibody (goat anti-rat; abcam 97054; Abcam, Cambridge, United Kingdom). Afterward, the cells were stained with the chromogen Permanent Red (Ref. K0640, DAKO GmbH, Jena, Germany) and counterstained with hematoxylin. For quantification, the total number of F4/80-positive cells (F4/80+) and the total number of hepatocytes were also counted in a blinded manner in 10 consecutive HPF at 400× magnification.

### Western blot analysis

The harvested liver tissue and HepG2 cells incubated with TNFα were further processed for protein isolation. For this purpose, the liver tissue and cells were homogenized in lysis buffer (10 mM Tris pH 7.5, 10 mM NaCl, 0.1 mM EDTA, 0.5% Triton-X100, 0.02% NaN3, and 0.2 mM PMSF, protease inhibitor cocktail), incubated for 30 min on ice, and centrifuged for 10 min at 4°C and 10,000 × *g*. Protein contents were assayed by using the bicinchoninic acid method (Pierce Biotechnology Inc., Thermo Fisher Scientific, Waltham, MA, United States), with 2.5% BSA (Pierce Biotechnology Inc., Thermo Fisher Scientific, Waltham, MA, United States) as the standard. On an 8% SDS gel (FGFR1c and pFGFR1c) and a 10% Mini-PROTEAN^®^ TGX Stain-FreeTM (Bio-Rad Laboratories, Munich, Germany) gel (β-klotho), 20 (liver tissue) or 10 (HepG2 cells) μg protein was separated. Mini-PROTEAN TGX gel was captured using the ChemiDoc XRS System (Bio-Rad Laboratories, Munich, Germany) before being transferred to a polyvinyldifluoride membrane (Immobilon-P; Millipore, Eschborn, Germany). After blockade with 2.5% BSA (Santa Cruz Biotechnology, Santa Cruz, CA, United States), membranes were incubated overnight at 4°C with a rabbit polyclonal anti-β-klotho (1:1.000, LSBioScience, Seattle, WA, United States), a rabbit polyclonal anti-pFGFR1c (Tyr653/654; 1:1.000, Cell Signaling Technology, Cambridge, United Kingdom), or a rabbit monoclonal anti-FGF21 [EPR8314(2), only HepG2 cells, 1:1.000, abcam, Cambridge, United Kingdom] antibody, respectively. Afterward, a secondary peroxidase-linked anti-rabbit antibody (β-klotho and pFGFR1c, 1:10.000; Cell Signaling Technology, Cambridge, United Kingdom) or only HepG2 cells (FGF21, 1:3.000) was applied. Protein expression was visualized by means of luminol-enhanced chemiluminescence (ECL plus; Amersham Pharmacia Biotech, Freiburg, Germany) and digitalized using the ChemiDoc™ XRS System (Bio-Rad Laboratories, Feldkirchen, Germany). Signals were densitometrically assessed (Quantity One; Bio-Rad Laboratories, Munich, Germany) and normalized either to the GAPDH signals (β-klotho and FGF21, HepG2 cells, and mouse monoclonal anti-β-GAPDH antibody; 1:20.000; Millipore, Eschborn, Germany, followed by secondary anti-mouse antibody, 1:40.000, Sigma Aldrich Corp., St. Louis, MO, United States) or to whole protein (β-klotho, liver tissue). To analyze the phosphorylation status of FGFR1c, signals of pFGFR1c were normalized to rabbit polyclonal anti-FGFR1c (clone D8E4, 1:1.000, Cell Signaling Technology, Cambridge, United Kingdom).

### Quantitative real-time polymerase chain reaction

Ribonucleic acid (RNA) isolation and transcription into cDNA were performed as already published (28). mRNA expression analyses were performed *via* quantitative real-time polymerase chain reaction (PCR) in a BioRad iQ5 Multicolor Real Time PCR Detection System (Conquer Scientific, San Diego, CA, United States) with an iQ™ SYBR^®^ Green Supermix (Bio-Rad Laboratories, Munich, Germany). Primer sequences are shown in Table 1. Measurement results are corrected against the housekeeping gene 40S ribosomal protein S18 (RPS18), and relative quantification was carried out *via* the 2^–ΔΔ*CT*^ method.

**TABLE 1:** Primers used for quantitative real-time polymerase chain reaction (PCR).

Transcript	Forward primer (5′–3′)	Reverse primer (5′–3′)
*fgf21*	GCTGTCTTCCTGCTGGGG	CCTGGTTTGGGGAGTCCTTC
*tnf*α	ACATTCGAGGCTCCAGT GAATTCGG	GGCAGGTCTACTTTGGAGT CATTGC
*il-1*β	CCCAAGCAATACCCAAAGAA	TTGTGAGGTGCTGATGTACCA
*il-6*	TCTGACCACAGTGAGGA ATGTCCAC	TGGAGTCACAGAAGGAGT GGCTAAG
*il-10*	GCCTTGCAGAAAAGAGAGCT	AAAGAAAGTCTTCACCTGGC
*rps18*	AGGATGTGAAGGATGGGAAG	TTGGATACACCCACAGTTCG

### Statistics

Statistical analysis was performed using GraphPad Prism 8.0.1 (GraphPad Software Inc., San Diego, CA, United States) as previously described by our group (28). Data from animals that died during the experimental period or showed abnormalities during organ removal were excluded from all analysis. For expressional analyses of liver FGF21, IL-1β, IL-6, IL-10, and TNFα, each *n* = 7 samples and for HDL, LDL, and cholesterol assays, each *n* = 5 samples were measured. The ROUT method based on the false discovery rate (*Q* = 0.01) was applied to remove outliers. All results are presented as mean ± standard deviation (SD), and statistical significance was set at *p* < 0.05. For further details, see figure legends.

To represent correlation between all observational parameters, Pearson correlation was performed by measuring linear dependence of two parameters. Clustering between the six groups is represented as a dot plot of principal component analysis (PCA). For the parameters TNFα, FGF21, and β-klotho expressions in the liver, all data from *n* = 84 mice are included. Correlation between all 26 parameters (for AST, ALT, insulin, and leptin, see Supplementary Figure 1) is shown in a heatmap, indicating a strong positive correlation by red color (1.00–0.70), a strong negative correlation by blue color (−0.70 to −1.00), and a moderate correlation by light colors (>0.40 or <-0.40) (35). The analysis was carried out in R (version 4.0.2, R studio, Boston, United States) *via* the prcomp method. Missing values were imputed ahead of analysis using the mean of the respective experimental group.

## Results

### Diet-induced obesity is attenuated by intervention strategies

**FIGURE 2:**
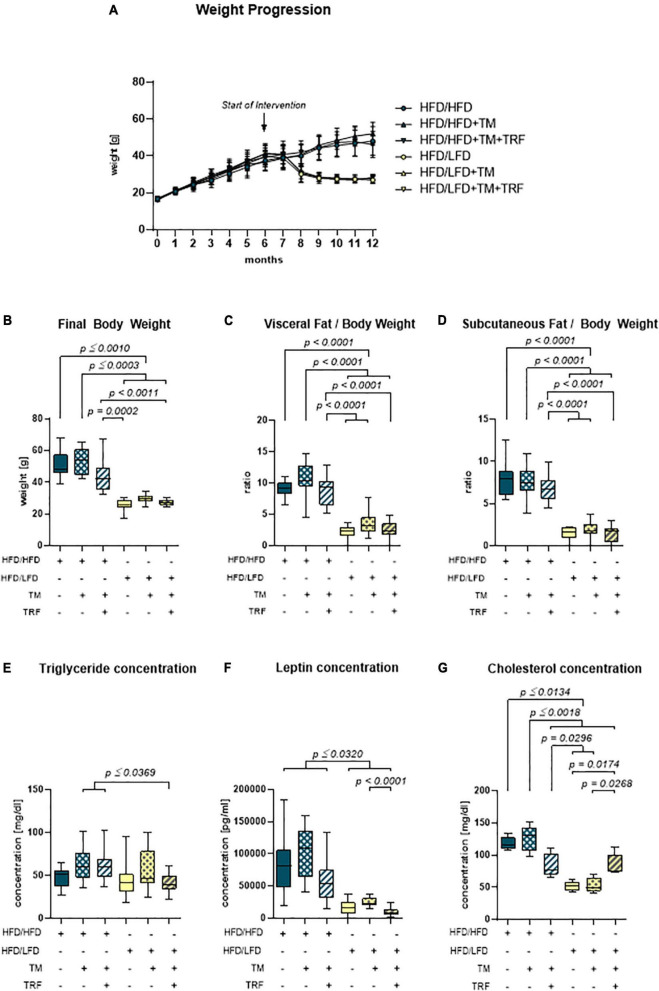
Body and fat composition. **(A)** Monthly weight progression with *n* = 90 mice at the beginning and *n* = 84 mice at the final time point. **(B)** Final body weights [g] before euthanasia (HFD/HFD: *n* = 13, HFD/HFD + TM: *n* = 13, HFD/HFD + TM + TRF: *n* = 15, HFD/LFD: *n* = 14, HFD/LFD + TM: *n* = 15, HFD/LFD + TM + TRF: *n* = 13; total *n* = 83). **(C)** Ratio of visceral body fat deposits to body weight. **(D)** Ratio of subcutaneous flanked fat deposits to body weight (**C,D** HFD/HFD: *n* = 13, HFD/HFD + TM: *n* = 13, HFD/HFD + TM + TRF: *n* = 15, HFD/LFD: *n* = 14, HFD/LFD + TM: *n* = 15, HFD/LFD + TM + TRF: *n* = 14; total *n* = 84). All HFD/LFD groups showed a significant fat loss with *p* < 0.0001 when compared to all three HFD/HFD groups, respectively [**A–D** from Power Guerra et al. (28)]. **(E)** Plasma triglyceride [mg/dL] (HFD/HFD: *n* = 13, HFD/HFD + TM: *n* = 13, HFD/HFD + TM + TRF: *n* = 15, HFD/LFD: *n* = 14, HFD/LFD + TM: *n* = 15, HFD/LFD + TM + TRF: *n* = 14; total *n* = 84). **(F)** Plasma leptin [pg/mL] (HFD/HFD: *n* = 13, HFD/HFD + TM: *n* = 13, HFD/HFD + TM + TRF: *n* = 14, HFD/LFD: *n* = 14, HFD/LFD + TM: *n* = 15, HFD/LFD + TM + TRF: *n* = 13; total *n* = 82) and **(G)** plasma cholesterol [mg/dL] (HFD/HFD: *n* = 5, HFD/HFD + TM: *n* = 5, HFD/HFD + TM + TRF: *n* = 5, HFD/LFD: *n* = 5, HFD/LFD + TM: *n* = 5, HFD/LFD + TM + TRF: *n* = 5; total *n* = 30). Blue dots and box plots indicate HFD groups, and yellow dots and box plots indicate dietary change to LFD. Table displays the individual groups, respectively. Table is read from top to bottom; “+” denotes implementation of a given diet or intervention and “−” its absence. Significance of differences between groups was tested with either the Kruskal–Wallis test, followed by Dunn’s *post hoc* test for multiple comparisons **(B)**, the Brown–Forsythe test, and Welch’s ANOVA with the Tamhane T2 *post hoc* test for multiple comparisons [**C:** *F* value (*F*) = 51.82, degree of freedom (DF) = 5; **D:** *F* = 66.19; DF = 5. **F:** *F* = 22.68, DF = 5], or by ordinary one-way ANOVA with Tukey’s *post hoc* test for multiple comparisons **(E)**. Data are presented as mean ± SD, and statistical significance was set at *p* < 0.05. HFD, high-fat diet; LFD, low-fat diet; TM, treadmill; TRF, time-restricted feeding.

Continuous administration of the HFD caused a large increase in body weight within the first 6 months (Figure 2A). After the introduction of the intervention approaches, such as LFD, TM training, and TRF, only the dietary change to LFD resulted in weight loss within a few weeks (Figure 2A, yellow vs. blue). Also, final body weight, and visceral and subcutaneous fat-to-body weight ratios were about 50% lower than those in all HFD/HFD groups (blue) (Figures 2B–D, yellow vs. blue). While in the HFD/LFD groups triglyceride concentrations were only decreased in tendency (Figure 2E), the concentrations of leptin and cholesterol were significantly reduced (yellow; Figures 2F,G).

### High-fat diet-induced non-alcoholic fatty liver disease is treatable by intervention strategies leading to reduced pro-inflammatory tumor necrosis factor alpha expression

**FIGURE 3:**
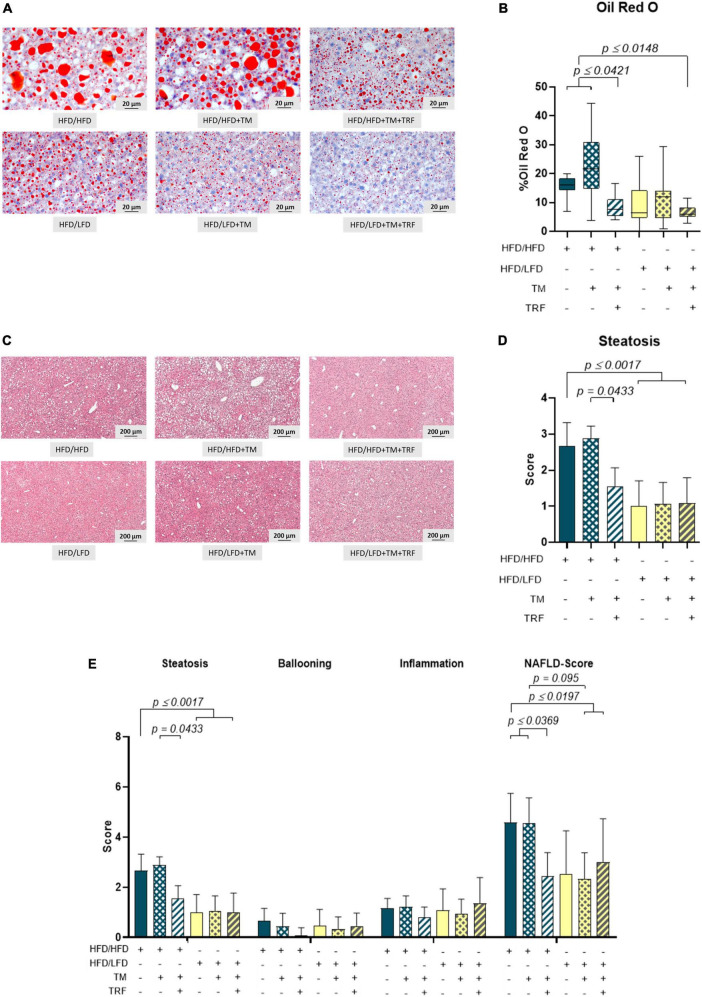
Representative images of Oil Red O **(A)** (400× magnification, scale bar represents 200 μm) and HE-stained liver specimen. **(C)** (50× magnification, scale bar represents 200 μm) and quantitative analysis of the Oil Red O-stained area in percentage **(B)** (HFD/HFD: *n* = 11, HFD/HFD + TM: *n* = 11, HFD/HFD + TM + TRF: *n* = 12, HFD/LFD: *n* = 13, HFD/LFD + TM: *n* = 15, HFD/LFD + TM + TRF: *n* = 10; total *n* = 72) and steatosis score **(D)** (HFD/HFD: *n* = 12, HFD/HFD + TM: *n* = 9, HFD/HFD + TM + TRF: *n* = 11, HFD/LFD: *n* = 13, HFD/LFD + TM: *n* = 15, HFD/LFD + TM + TRF: *n* = 11; total *n* = 71). Assessments of scores for steatosis, ballooning, and inflammation, as well as calculation of NAS for the groups, are represented in panel **E**. Blue dots and box plots indicate HFD groups, and yellow dots and box plots indicate dietary change to LFD. Table displays the individual groups, respectively. Table is read from top to bottom; “+” denotes implementation of a given diet or intervention and “−” its absence. Significance of differences between groups was tested with either the Kruskal–Wallis test, followed by Dunn’s *post hoc* test for multiple comparisons **(B)**, the Brown–Forsythe test, and Welch’s ANOVA with the Tamhane T2 *post hoc* test for multiple comparisons [**D:** *F* value (*F*) = 8.297, degree of freedom (DF) = 5, or by ordinary one-way ANOVA with Tukey’s *post hoc* test for multiple comparisons (**E:** *F* = 9.765, DF = 5)]. Data are presented as mean ± SD, and statistical significance was set at *p* < 0.05. HFD, high-fat diet; LFD, low-fat diet; TM, treadmill; TRF, time-restricted feeding.

**FIGURE 4:**
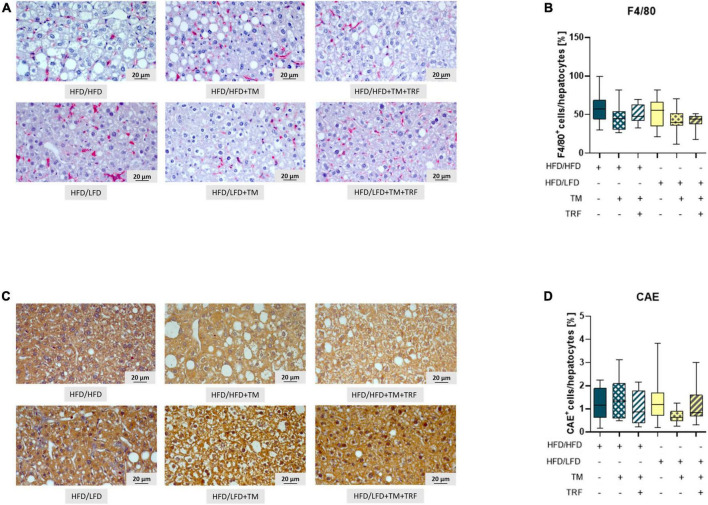
Representative images (both at 400× magnification, scale bar representing 20 μm) of F4/80 **(A)** as well as CAE-stained livers **(C)** and quantitative analysis of F4/80+ (**B:** HFD/HFD: *n* = 12, HFD/HFD + TM: *n* = 10, HFD/HFD + TM + TRF: *n* = 12, HFD/LFD: *n* = 13, HFD/LFD + TM: *n* = 15 or HFD/LFD + TM + TRF: *n* = 11; total *n* = 73) as well as CAE+ (**D:** HFD/HFD: *n* = 12, HFD/HFD + TM: *n* = 9, HFD/HFD + TM + TRF: *n* = 12, HFD/LFD: *n* = 13, HFD/LFD + TM: *n* = 13, HFD/LFD + TM + TRF: *n* = 11; total *n* = 70). Blue dots and box plots indicate HFD groups, and yellow dots and box plots indicate dietary change to LFD. Table displays the individual groups, respectively. Table is read from top to bottom; “+” denotes implementation of a given diet or intervention and “−” its absence. Significance of differences between groups was tested with either the Kruskal–Wallis test, followed by Dunn’s *post hoc* test for multiple comparisons **(B)** or the Brown–Forsythe test, and Welch’s ANOVA with the Tamhane T2 *post hoc* test for multiple comparisons **(D)** [*F* value (*F*) = 1.313, degree of freedom (DF) = 5]. Data are presented as mean ± SD, and statistical significance was set at *p* < 0.05. HFD, high-fat diet; LFD, low-fat diet; TM, treadmill; TRF, time-restricted feeding.

**FIGURE 5:**
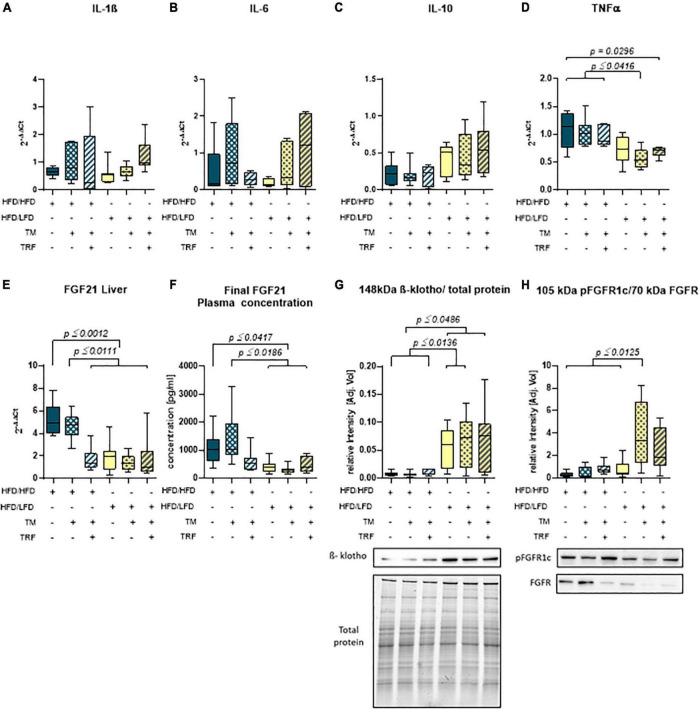
Hepatic mRNA expression of IL-1β **(A)** (HFD/HFD: *n* = 6, HFD/HFD + TM: *n* = 7, HFD/HFD + TM + TRF: *n* = 7, HFD/LFD: *n* = 7, HFD/LFD + TM: *n* = 7, HFD/LFD + TM + TRF: *n* = 7; total *n* = 41), IL-6 **(B)** (HFD/HFD: *n* = 6, HFD/HFD + TM: *n* = 7, HFD/HFD + TM + TRF: *n* = 7, HFD/LFD: *n* = 6, HFD/LFD + TM: *n* = 7, HFD/LFD + TM + TRF: *n* = 7; total *n* = 40), IL-10 **(C)** (HFD/HFD: *n* = 7, HFD/HFD + TM: *n* = 7, HFD/HFD + TM + TRF: *n* = 6, HFD/LFD: *n* = 7, HFD/LFD + TM: *n* = 7, HFD/LFD + TM + TRF: *n* = 7; total *n* = 41), TNFα **(D)** (HFD/HFD: *n* = 6, HFD/HFD + TM: *n* = 7, HFD/HFD + TM + TRF: *n* = 7, HFD/LFD: *n* = 7, HFD/LFD + TM: *n* = 7, HFD/LFD + TM + TRF: *n* = 7; total *n* = 41), plasma concentration **(E)** (HFD/HFD: *n* = 13, HFD/HFD + TM: *n* = 13, HFD/HFD + TM + TRF: *n* = 15, HFD/LFD: *n* = 12, HFD/LFD + TM: *n* = 14, HFD/LFD + TM + TRF: *n* = 11; total *n* = 78), and hepatic mRNA expression **(F)** (HFD/HFD: *n* = 6, HFD/HFD + TM: *n* = 6, HFD/HFD + TM + TRF: *n* = 7, HFD/LFD: *n* = 7, HFD/LFD + TM: *n* = 7, HFD/LFD + TM + TRF: *n* = 7; total *n* = 40) of FGF21. Data presented as 2^– ΔΔ*Ct*^ values determined by quantitative real-time PCR. Quantitative analysis of hepatic protein expression of β-klotho **(G)** (HFD/HFD: *n* = 12, HFD/HFD + TM: *n* = 9, HFD/HFD + TM + TRF: *n* = 10, HFD/LFD: *n* = 12, HFD/LFD + TM: *n* = 15, HFD/LFD + TM + TRF: *n* = 11; total *n* = 69) and phosphorylated form of the FGF receptor **(H)** (HFD/HFD: *n* = 12, HFD/HFD + TM: *n* = 10, HFD/HFD + TM + TRF: *n* = 10, HFD/LFD: *n* = 9, HFD/LFD + TM: *n* = 10, HFD/LFD + TM + TRF: *n* = 8; total *n* = 59) both with representative Western blots. Signals were normalized either to total protein (β-klotho) or to the non-phosphorylated FGF receptor. Blue dots and box plots indicate HFD groups, and yellow dots and box plots indicate dietary change to LFD. Table displays the individual groups, respectively. Table is read from top to bottom, and “+” denotes implementation of a given diet or intervention and “−” its absence. Significance of differences between groups was tested with either the Kruskal–Wallis test, followed by Dunn’s *post hoc* test for multiple comparisons **(B)**, Brown–Forsythe, and Welch’s ANOVA with the Tamhane T2 *post hoc* test for multiple comparisons [**A:** *F* value (*F*) = 1.165, degree of freedom (DF) = 5; **D:** *F* = 5.742, DF = 5. **E:** *F* = 11.65, DF = 5, **F:** *F* = 9.902, DF = 5 **G:** *F* = 10.33; DF = 5, **H:** *F* = 9.214; DF = 5], or by ordinary one-way ANOVA with Tukey’s *post hoc* test for multiple comparisons **(C)**. Data are presented as mean ± SD, and statistical significance was set at *p* < 0.05. HFD, high-fat diet; LFD, low-fat diet; TM, treadmill; TRF, time-restricted feeding.

Accordingly, liver fat content, evaluated by Oil Red O staining, was found to be significantly diminished after dietary change (Figures 3A,B), which was also reflected by a significantly lower steatosis score (Figures 3C,D). Notably, livers of the HFD mice receiving TM and TRF displayed almost the same histological changes as observed in all LFD groups. In particular, Oil Red O staining showed a significant reduction in liver lipid in the HFD/HFD + TM + TRF group compared to the HFD/HFD group (Figure 3B). Although the parameters of hepatocellular ballooning and lobular inflammation were largely unchanged upon interventions, the NAFLD score was significantly decreased in all groups with dietary change, and in particular upon TM and TRF in the HFD group (Figure 3E). Despite the strong decrease in the lipid content after dietary change, there was no reduction in the number of resident macrophages and granulocytes in the liver, as indicated by no significant differences in the number of F4/80 (Figures 4A,B) and CAE (Figures 4C,D)-positive cells between the individual experimental groups. Consistent with this, mRNA expressions of the pro-inflammatory cytokines IL-1β and IL-6 were also nearly unchanged (Figures 5A,B), but the anti-inflammatory cytokine IL-10 showed tendencies to be increased mainly upon dietary change (Figure 5C). Noteworthy, the mRNA expression of TNFα was significantly decreased in all LFD vs. HFD groups (Figure 5D).

### Reduced tumor necrosis factor alpha expression is accompanied by restoration of an fibroblast growth factor 21 sensitive state

**FIGURE 6:**
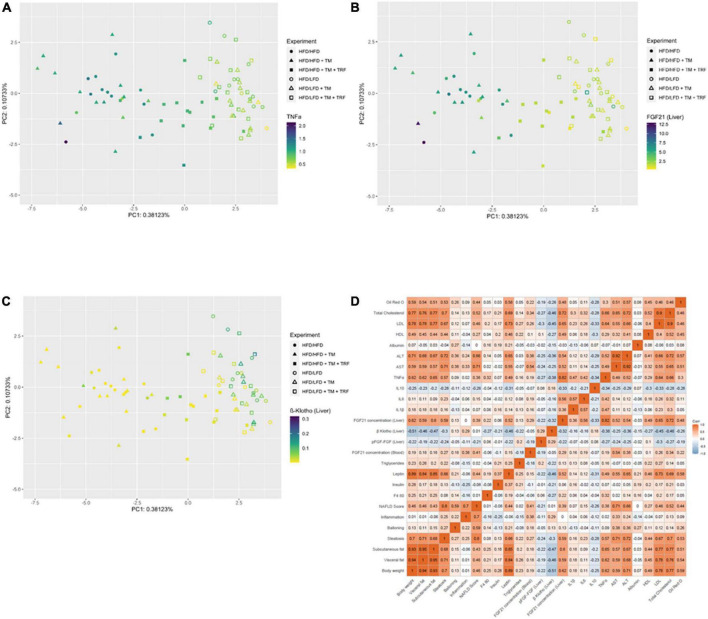
Dot plot of principal component analysis (PCA) for TNFα **(A)**, FGF21 in the liver **(B)**, and β-klotho in the liver **(C)** and correlation (Pearson, two-sided test, statistical significance was set at *p* < 0.05) represented as a multidimensional heatmap **(D)**. All parameters from previously acquired experiments were further used for PCA construction. TNFα (**A:** HFD/HFD *n* = 13, HFD/HFD + TM *n* = 13, HFD/HFD + TM + TRF *n* = 15, HFD/LFD *n* = 14, HFD/LFD + TM *n* = 15, HFD/LFD + TM + TRF *n* = 14; total *n* = 84), FGF21 liver (**B:** HFD/HFD *n* = 13, HFD/HFD + TM *n* = 13, HFD/HFD + TM + TRF *n* = 15, HFD/LFD *n* = 14, HFD/LFD + TM *n* = 15, HFD/LFD + TM + TRF *n* = 14; total *n* = 84), and β-klotho liver (**C:** HFD/HFD *n* = 13, HFD/HFD + TM *n* = 13, HFD/HFD + TM + TRF *n* = 15, HFD/LFD *n* = 14, HFD/LFD + TM *n* = 15, HFD/LFD + TM + TRF *n* = 14; total *n* = 84). In panels **A–C**, filled circles, triangles, and squares represent groups with HFD, and non-filled indicate dietary change to LFD. In panel **D**, red color indicates positive correlation, blue color indicates negative correlation, while white indicates no correlation. HFD, high-fat diet; LFD, low-fat diet; TM, treadmill; TRF, time-restricted feeding.

Parallel to the reduction of TNFα, a significant decrease in the FGF21 concentration in plasma (Figure 5F) was observed. This was in line with the significant reduction of hepatic FGF21 mRNA expression (Figure 5E), whereby HFD mice receiving additional TM and TRF showed similar values, indicating that not only dietary change but also physical activity and intermittent fasting may trigger FGF21 levels. Of particular interest was the significant increase in hepatic β-klotho expression in all dietary change groups (Figure 5G), which was also associated with increased phosphorylation of FGFR1c, although this was only seen in the LFD groups with additional TM and TRF (Figure 5H). These results now suggest that especially with the dietary change, FGF21 sensitivity could be restored, that is, reduced FGF21 expression and increased expression of its receptors. The statistical results were confirmed by PCA (Figure 6). PCA showed for hepatic TNFα (A), FGF21 (B), and β-klotho (C) that the LFD groups clustered mainly on one side and the HFD groups on the other. In addition to this, the trained HFD group with additional intermittent fasting clustered more with the LFD groups. A further consideration of the correlation analysis (Figure 6D) showed that TNFα correlated with parameters, such as body weight, percentage of adipose tissue, and liver fat content (on average with *r* = 0.6; all *p* ≤ 0.05). Of particular interest is that the NAFLD score positively correlated with hepatic TNFα (*r* = 0.38; *p* ≤ 0.05), and particularly with systemic FGF21 (*r* = 0.41; *p* ≤ 0.05).

**FIGURE 7:**
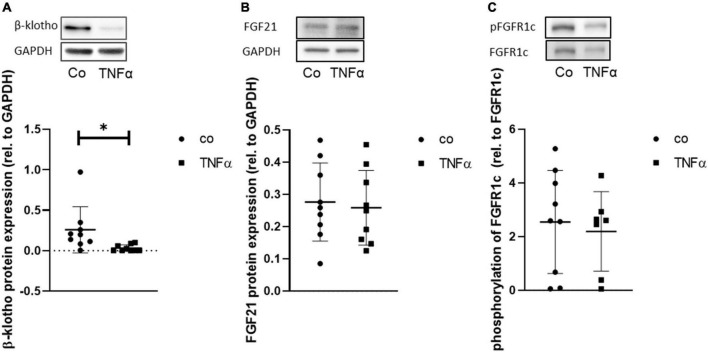
Representative Western blot and quantitative analysis of protein expression of β-klotho **(A)** and of FGF21, **(B)** and phosphorylation of FGFR1c **(C)** in HepG2 cells upon incubation of TNF for 24 h. Signals were normalized to GAPDH or FGFR1c, respectively. Data are presented as mean ± SD; *n* = 9 independent experiments; unpaired Student’s *t*-test, *p < 0.05 vs. control (Co).

Furthermore, the degree of dependency between TNFα and FGF21 sensitivity was investigated by correlation analysis (Figure 6D). We found that hepatic β-klotho correlated negatively with hepatic TNFα (*r* = −0.38; *p* ≤ 0.05) and with hepatic FGF21 (*r* = −0.32; *p* ≤ 0.05), while hepatic FGF21 correlated strongly positively with TNFα (*r* = 0.82; *p* ≤ 0.05). This finding was partly supported by the *in vitro* analysis in HepG2 cells, showing that TNFα is indeed able to significantly reduce β-klotho expression (Figure 7A), whereas the phosphorylation of FGFR1c and protein expression of FGF21 was almost unchanged (Figures 7B,C).

## Discussion and conclusion

The main finding of the study was that reversion of NAFLD is achieved not only by dietary change but also by continued HFD combined with exercise and intermittent fasting. In addition, by observing decreased TNFα and FGF21 expression with concomitant increased β-klotho expression and a strong negative correlation between TNFα and β-klotho expression, we conclude that NAFLD recovery is associated with reversal of a TNFα-triggered FGF21-resistant state or desensitization.

For the treatment of NAFLD, next to bariatric surgery and pharmacological approaches, a less invasive method, namely, dietary change, is also feasible in some obese cases (36, 37). Thus, reduced calorie intake by lowering the fat content is recommended for sustainable weight loss (36) and thus for NAFLD recovery. As proof of principal, this was confirmed in the present study as indicated by the lowered liver fat content and steatosis score. Supporting this, our correlation analyses showed that a decrease in body weight and subcutaneous, as well as visceral fat, is associated with a reduced NAFLD score. In addition to dietary modification, physical activity is another modality for weight reduction in NAFLD therapy (38). However, in the present study, independent of LFD or HFD, no additional benefit was reached with treadmill exercise, as also shown by Ringseis et al. (39). This may be due to the frequency of training as daily training is recommended for a reduction in body weight and thus for NAFLD therapy (37). Furthermore, the currently widespread intermittent fasting may have beneficial effects on NAFLD, especially since is knowing that this contributes to weight loss in obesity (40). Contrary to expectation, only the HFD mice receiving a time-restricted feeding additional to exercise revealed a reduced NAFLD score. As exercise alone did not benefit in NAFLD recovery, we conclude that in particular intermittent fasting in continued HFD is responsible for the observed effect. Similarly, cholesterol concentration was reduced only in the HFD group that received intermittent fasting in addition to exercise, which contradicts the study by Swift et al. (41), who showed that exercise alone reduced blood lipid levels. However, the extent to which the two interventions—treadmill training and intermittent feeding—influence each other in the context of a continued HFD and possibly overshadow any protective effect of training by intermittent fasting or vice versa can only be speculated. Nevertheless, the beneficial effect of time-restricted feeding is in line with the study of Chaix et al. (40) showing that body weight and body fat composition were markedly reduced in the HFD mice, which may be causative for recovery of NAFLD, as shown in the present study.

Obesity *per se* is often associated with increasing inflammatory mediators, such as TNFα (8). In turn, the reversion of NAFLD correlated positively with the reduction of TNFα expression, which persisted not only in dietary change but also in the HFD group receiving treadmill training and time-restricted feeding. TNFα has the ability to decrease the expression of β-klotho but not the phosphorylation of FGFR1c, as shown by Diaz-Delfin et al. (24) in adipocytes. This finding was underlined by the current study in hepatocytes, further highlighting the stronger role of β-klotho in FGF21 signaling. This statement is further supported when considering the groups with NAFLD recovery, where there was a stronger negative correlation of TNFα with β-klotho than with pFGFR1c, confirming the assumption that TNFα may alter FGF21 responsiveness, mainly *via* β-klotho. Although no increase in FGF21 expression could be detected after TNFα application *in vitro*, hepatic FGF21 expression, especially the circulating FGF21 plasma concentration, was decreased in mice that received dietary change in general. Interestingly, the exercising HFD group receiving additional time-restricted feeding also showed reduced FGF21 levels, suggesting that not only dietary change alone but also intermittent fasting may recover from obesity-induced FGF21 desensitization (20). This is also confirmed by the distribution of TNFα, FGF21, and β-klotho data points in the PCA plots, as indicated by clustering not only within the LFD groups but also within the treadmill training HFD group, which additionally underwent intermittent fasting. Accordingly, intermittent fasting has also been described to protect against consequences of obesity (27, 40), including the FGF21-resistant state (21). Since FGF21 has a circadian rhythm that is disrupted by a high-fat diet, intermittent fasting is thought to rebalance the oscillation of FGF21 by coupling food intake in a time-of-day-dependent manner (42, 43) and thus counteracts obesity. Moreover, Geng et al. (26) showed that physical activity can decrease FGF21 expression and restore FGF21 sensitivity in obese mice and rebalance the metabolic interaction between the adipose tissue, liver, and skeletal muscle. However, this was not observed in the current study because exercise alone in the LFD as well as in the HFD group did not provide any additional benefit—neither in ameliorating NAFLD nor in reversing FGF21 sensitivity. Nevertheless, correlation analysis revealed, independent of any interventions, that with enhanced NAFLD recovery, TNFα expression was decreased, leading to increased FGF21 sensitivity expressed as an increase in β-klotho and FGFR1c expression with concomitantly reduced FGF21 levels. Thus, we conclude that hepatic FGF21 resistance or desensitization is most likely TNFα-dependent. Moreover, it was also observed that mainly circulating FGF21 correlates with the NAFLD score, suggesting a potential dependency between treated NAFLD and FGF21. This finding could provide a basis for considering non-invasive determination of plasma FGF21 as a possible marker to monitor NAFLD activity. This approach might have a high translational potential in treatment of NAFLD in obese patients.

## Data availability statement

The datasets presented in this study can be found in online repositories. The names of the repository/repositories and accession number(s) can be found in the article/Supplementary material.

## Ethics statement

This animal study was reviewed and approved by Landesamt für Landwirtschaft, Lebensmittelsicherheit und Fischerei (LALLF) of the state Mecklenburg-Western Pomerania (LALLF M-V/TSD/7221.3-2-001/18, approved on March 1, 2018).

## Author contributions

AK: conceptualization, funding acquisition, and project administration. NPG: data curation, investigation, and validation. KL and LM: formal analysis. NPG, KL, LM, and DB: methodology. BV: resources. AK and BV: supervision. KL: visualization. AK, NPG, KL, and LM: roles and writing—original draft. DJ, SS, KP, BV, and HG: writing—review and editing. All authors contributed to the article and approved the submitted version.

## References

[1] Kaplan NM. The deadly quartet. Upper-body obesity, glucose intolerance, hypertriglyceridemia, and hypertension. *Arch Int Med.* (1989) 149:1514–20. 10.1001/archinte.149.7.1514 2662932

[2] Beilby J. Definition of metabolic syndrome: report of the national heart, lung, and blood institute/american heart association conference on scientific issues related to definition. *Clin Biochem Rev.* (2004) 25:195–8.

[3] Gogia A Agarwal PK. Metabolic syndrome. *Indian J Med Sci.* (2006) 60:72–81. 16505579

[4] Marchesini G Brizi M Bianchi G Tomassetti S Bugianesi E Lenzi M Nonalcoholic fatty liver disease: a feature of the metabolic syndrome. *Diabetes.* (2001) 50:1844–50. 10.2337/diabetes.50.8.1844 11473047

[5] Schattenberg JM Schuppan D. Nonalcoholic steatohepatitis: the therapeutic challenge of a global epidemic. *Curr Opin Lipidol.* (2011) 22:479–88. 10.1097/MOL.0b013e32834c7cfc 22002020

[6] Hotamisligil GS. Inflammation and metabolic disorders. *Nature.* (2006) 444:860–7. 10.1038/nature05485 17167474

[7] Lumeng CN Saltiel AR. Inflammatory links between obesity and metabolic disease. *J Clin Invest.* (2011) 121:2111–7. 10.1172/JCI57132 21633179 PMC3104776

[8] Hotamisligil GS. Inflammation, metaflammation and immunometabolic disorders. *Nature.* (2017) 542:177–85. 10.1038/nature21363 28179656

[9] Juge-Aubry CE Henrichot E Meier CA. Adipose tissue: a regulator of inflammation. *Best Pract Res Clin Endocrinol Metab.* (2005) 19:547–66. 10.1016/j.beem.2005.07.009 16311216

[10] Wellen KE Hotamisligil GS. Obesity-induced inflammatory changes in adipose tissue. *J Clin Invest.* (2003) 112:1785–8. 10.1172/JCI20514 14679172 PMC297006

[11] Kim KA Gu W Lee IA Joh EH Kim DH. High fat diet-induced gut microbiota exacerbates inflammation and obesity in mice via the TLR4 signaling pathway. *PLoS One.* (2012) 7:e47713. 10.1371/journal.pone.0047713 23091640 PMC3473013

[12] Wu Z Xu J Tan J Song Y Liu L Zhang F Mesenteric adipose tissue B lymphocytes promote local and hepatic inflammation in non-alcoholic fatty liver disease mice. *J Cell Mol Med.* (2019) 23:3375–85. 10.1111/jcmm.14232 30772951 PMC6484337

[13] Koyama Y Brenner DA. Liver inflammation and fibrosis. *J Clin Invest.* (2017) 127:55–64. 10.1172/JCI88881 28045404 PMC5199698

[14] Saltiel AR Olefsky JM. Inflammatory mechanisms linking obesity and metabolic disease. *J Clin Invest.* (2017) 127:1–4. 10.1172/JCI92035 28045402 PMC5199709

[15] Kharitonenkov A Shiyanova TL Koester A Ford AM Micanovic R Galbreath EJ FGF-21 as a novel metabolic regulator. *J Clin Invest.* (2005) 115:1627–35. 10.1172/JCI23606 15902306 PMC1088017

[16] Coskun T Bina HA Schneider MA Dunbar JD Hu CC Chen Y Fibroblast growth factor 21 corrects obesity in mice. *Endocrinology.* (2008) 149:6018–27. 10.1210/en.2008-0816 18687777

[17] Luo Y Ye S Li X Lu W. Emerging structure-function paradigm of endocrine FGFs in metabolic diseases. *Trends Pharmacol Sci.* (2019) 40:142–53. 10.1016/j.tips.2018.12.002 30616873

[18] Domouzoglou EM Maratos-Flier E. Fibroblast growth factor 21 is a metabolic regulator that plays a role in the adaptation to ketosis. *Am J Clin Nutr.* (2011) 93:901S–5S. 10.3945/ajcn.110.001941 21346090 PMC3057552

[19] Zhang Y Xie Y Berglund ED Coate KC He TT Katafuchi T The starvation hormone, fibroblast growth factor-21, extends lifespan in mice. *Elife.* (2015) 1:e00065. 10.7554/eLife.00065 23066506 PMC3466591

[20] Gallego-Escuredo JM Gómez-Ambrosi J Catalan V Domingo P Giralt M Frühbeck G Opposite alterations in FGF21 and FGF19 levels and disturbed expression of the receptor machinery for endocrine FGFs in obese patients. *Int J Obesity.* (2015) 39:121–9. 10.1038/ijo.2014.76 24813368

[21] Fisher FM Chui PC Antonellis PJ Bina HA Kharitonenkov A Flier JS Obesity is a fibroblast growth factor 21 (FGF21)-resistant state. *Diabetes.* (2010) 59:2781–9. 10.2337/db10-0193 20682689 PMC2963536

[22] Kälin S Heppner FL Bechmann I Prinz M Tschöp MH Yi CX. Hypothalamic innate immune reaction in obesity. *Nat Rev Endocrinol.* (2015) 11:339–51. 10.1038/nrendo.2015.48 25824676

[23] Markan KR. Defining “FGF21 Resistance” during obesity: controversy, criteria and unresolved questions. *F1000Res.* (2018) 7:289. 10.12688/f1000research.14117.1 29983922 PMC6020717

[24] Díaz-Delfín J Hondares E Iglesias R Giralt M Caelles C Villarroya F. TNF-α represses β-Klotho expression and impairs FGF21 action in adipose cells: involvement of JNK1 in the FGF21 pathway. *Endocrinology.* (2012) 153:4238–45. 10.1210/en.2012-1193 22778214

[25] Kruse R Vienberg SG Vind BF Andersen B Højlund K. Effects of insulin and exercise training on FGF21, its receptors and target genes in obesity and type 2 diabetes. *Diabetologia.* (2017) 60:2042–51. 10.1007/s00125-017-4373-5 28721439

[26] Geng L Liao B Jin L Huang Z Triggle CR Ding H Exercise alleviates obesity-induced metabolic dysfunction via enhancing FGF21 sensitivity in adipose tissues. *Cell Rep.* (2019) 26:2738–52.e4. 10.1016/j.celrep.2019.02.014 30840894

[27] Chaix A Lin T Le HD Chang MW Panda S. Time-restricted feeding prevents obesity and metabolic syndrome in mice lacking a circadian clock. *Cell Metab.* (2019) 29:303–19.e4. 10.1016/j.cmet.2018.08.004 30174302 PMC7751278

[28] Power Guerra N Parveen A Bühler D Brauer DL Müller L Pilz K Fibroblast growth factor 21 as a potential biomarker for improved locomotion and olfaction detection ability after weight reduction in obese mice. *Nutrients.* (2021) 13:2916. 10.3390/nu13092916 34578793 PMC8470262

[29] Pietiläinen KH Kaprio J Borg P Plasqui G Yki-Järvinen H Kujala UM Physical inactivity and obesity: a vicious circle. *Obesity.* (2008) 16:409–14. 10.1038/oby.2007.72 18239652 PMC2249563

[30] Ferreira JC Rolim NP Bartholomeu JB Gobatto CA Kokubun E Brum PC. Maximal lactate steady state in running mice: effect of exercise training. *Clin Exp Pharmacol Physiol.* (2007) 34:760–5. 10.1111/j.1440-1681.2007.04635.x 17600553

[31] Guy CS Wang J Michalak TI. Hepatocytes as cytotoxic effector cells can induce cell death by CD95 ligand-mediated pathway. *Hepatology.* (2006) 43:1231–40. 10.1002/hep.21201 16729304

[32] Kleiner DE Brunt EM Van Natta M Behling C Contos MJ Cummings OW Nonalcoholic steatohepatitis clinical research network. Design and validation of a histological scoring system for nonalcoholic fatty liver disease. *Hepatology.* (2005) 41:1313–21. 10.1002/hep.20701 15915461

[33] Power Guerra N Müller L Pilz K Glatzel A Jenderny D Janowitz D Dietary-induced low-grade inflammation in the liver. *Biomedicines.* (2020) 8:587. 10.3390/biomedicines8120587 33317065 PMC7763065

[34] Liebig M Hassanzada A Kämmerling M Genz B Vollmar B Abshagen K. Microcirculatory disturbances and cellular changes during progression of hepatic steatosis to liver tumors. *Exp Biol Med.* (2018) 243:1–12. 10.1177/1535370217738730 29065724 PMC5788156

[35] Akoglu H. User’s guide to correlation coefficients. *Turk J Emerg Med.* (2018) 18:91–3. 10.1016/j.tjem.2018.08.001 30191186 PMC6107969

[36] Romero-Gómez M Zelber-Sagi S Trenell M. Treatment of NAFLD with diet, physical activity and exercise. *J Hepatol.* (2017) 67:829–46. 10.1016/j.jhep.2017.05.016 28545937

[37] Mantovani A Dalbeni A. Treatments for NAFLD: state of Art. *Int J Mol Sci.* (2021) 22:2350. 10.3390/ijms22052350 33652942 PMC7956331

[38] Nseir W Hellou E Assy N. Role of diet and lifestyle changes in nonalcoholic fatty liver disease. *World J Gastroenterol.* (2014) 20:9338–44. 10.3748/wjg.v20.i28.9338 25071328 PMC4110565

[39] Ringseis R Mooren FC Keller J Couturier A Wen G Hirche F Regular endurance exercise improves the diminished hepatic carnitine status in mice fed a high-fat diet. *Mol Nutr Food Res.* (2011) 55(Suppl. 2):S193–202. 10.1002/mnfr.201100040 21770048

[40] Chaix A Zarrinpar A Miu P Panda S. Time-restricted feeding is a preventative and therapeutic intervention against diverse nutritional challenges. *Cell Metab.* (2014) 20:991–1005. 10.1016/j.cmet.2014.11.001 25470547 PMC4255155

[41] Swift DL Houmard JA Slentz CA Kraus WE. Effects of aerobic training with and without weight loss on insulin sensitivity and lipids. *PLoS One.* (2018) 13:e0196637. 10.1371/journal.pone.0196637 29775461 PMC5959186

[42] Hatori M Vollmers C Zarrinpar A DiTacchio L Bushong EA Gill S Time-restricted feeding without reducing caloric intake prevents metabolic diseases in mice fed a high-fat diet. *Cell Metab.* (2012) 15:848–60. 10.1016/j.cmet.2012.04.019 22608008 PMC3491655

[43] Chapnik N Genzer Y Froy O. Relationship between FGF21 and UCP1 levels under time-restricted feeding and high-fat diet. *J Nutr Biochem.* (2017) 40:116–21. 10.1016/j.jnutbio.2016.10.017 27883936

