# Supplementary material for: The effect of different weight loss strategies to treat non-alcoholic fatty liver disease focusing on fibroblast growth factor 21
Source: Front Nutr. 2022 Aug 10;9:935805. doi: 10.3389/fnut.2022.935805 (PMC9399780; doi:10.3389/fnut.2022.935805)
Supplement: Supplementary file 2 [file Data_Sheet_1.docx]

Supplementary Material

# Supplementary Data

**ImageJ Code S1 for Oil Red O**

// Color Thresholder 1.52v

// Autogenerated macro, single images only!

min=newArray(3);

max=newArray(3);

filter=newArray(3);

a=getTitle();

run("RGB Stack");

run("Convert Stack to Images");

selectWindow("Red");

rename("0");

selectWindow("Green");

rename("1");

selectWindow("Blue");

rename("2");

min[0]=0;

max[0]=255;

filter[0]="pass";

min[1]=0;

max[1]=255;

filter[1]="pass";

min[2]=188;

max[2]=255;

filter[2]="pass";

for (i=0;i<3;i++){

selectWindow(""+i);

setThreshold(min[i], max[i]);

run("Convert to Mask");

if (filter[i]=="stop") run("Invert");

}

imageCalculator("AND create", "0","1");

imageCalculator("AND create", "Result of 0","2");

for (i=0;i<3;i++){

selectWindow(""+i);

close();

}

selectWindow("Result of 0");

close();

selectWindow("Result of Result of 0");

rename(a);

// Colour Thresholding-------------

run("8-bit");

run("Create Selection");

selectWindow(imgName);

run("Restore Selection");

run("Set Measurements...", "area display redirect=None decimal=3");

run("Measure");

run("Clear", "slice")

## Supplementary Figures

**
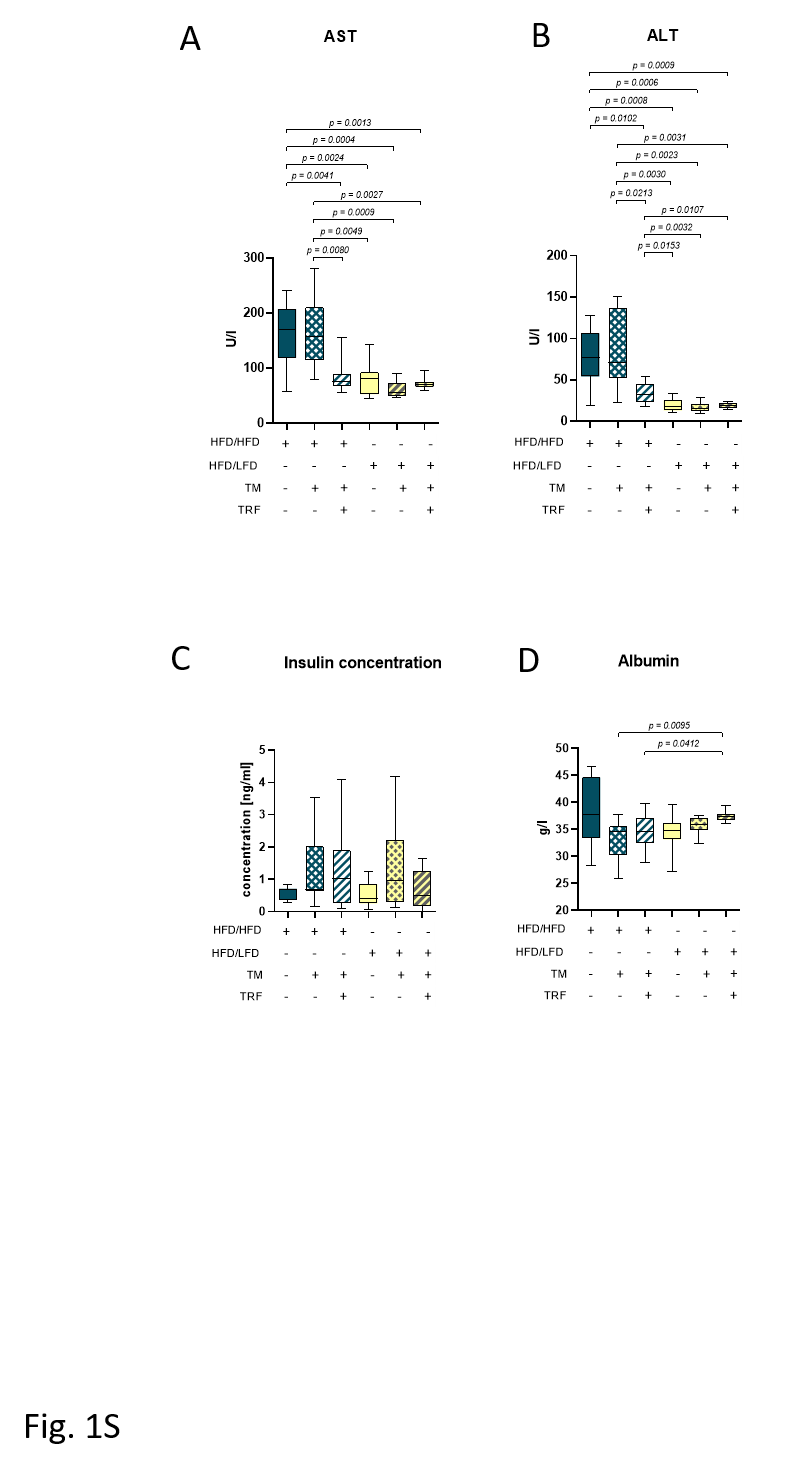
**

**Supplementary Figure 1.** Figure S1 (A) Final plasma AST [U/I] (HFD/HFD: n = 12, HFD/HFD+TM: n = 12, HFD/HFD+TM+TRF: n = 12, HFD/LFD: n = 13, HFD/LFD+TM: n = 14, HFD/LFD+TM+TRF: n = 10; total n = 73) (B) Final plasma ALT [U/I] (HFD/HFD: n = 12, HFD/HFD+TM: n = 12, HFD/HFD+TM+TRF: n = 12, HFD/LFD: n = 12, HFD/LFD+TM: n = 14, HFD/LFD+TM+TRF: n = 9; total n = 71) (C) Final insulin concentration [ng/ml] (HFD/HFD: n = 8, HFD/HFD+TM: n = 9, HFD/HFD+TM+TRF: n = 11, HFD/LFD: n = 13, HFD/LFD+TM: n = 15, HFD/LFD+TM+TRF: n = 9; total n = 65) (D) Final plasma Albumin [g/l] (HFD/HFD: n = 12, HFD/HFD+TM: n = 12, HFD/HFD+TM+TRF: n = 13, HFD/LFD: n = 13, HFD/LFD+TM: n = 14, HFD/LFD+TM+TRF: n = 11; total n = 75). Blue dots and box plots indicate HFD groups, yellow dots and box plots indicate diet change to LFD. The table below the figure displays the individual groups, respectively. Table is read from top to bottom, where ‘+' denotes a diet or intervention, whereas '-' does not refer to this parameter. Significance of differences between groups was tested with either Kruskal-Wallis test followed by Dunn’s post hoc test for multiple comparisons (C, D), Brown-Forsythe and Welch’s ANOVA with Tamhane T2 post hoc test for multiple comparisons (A: F value (F) = 20.35, Degree of Freedom (DF) = 5; B: F = 22.80 DF = 5. Data are presented as mean ± SD and statistical significance was set at p < 0.05. Abbreviations: HFD: high-fat diet, LFD: low-fat diet, TM: treadmill, TRF: time-restricted feeding.
