# Supplementary figures and images for: The effect of different weight loss strategies to treat non-alcoholic fatty liver disease focusing on fibroblast growth factor 21
Source: Front Nutr. 2022 Aug 10;9:935805. doi: 10.3389/fnut.2022.935805 (PMC9399780; doi:10.3389/fnut.2022.935805)

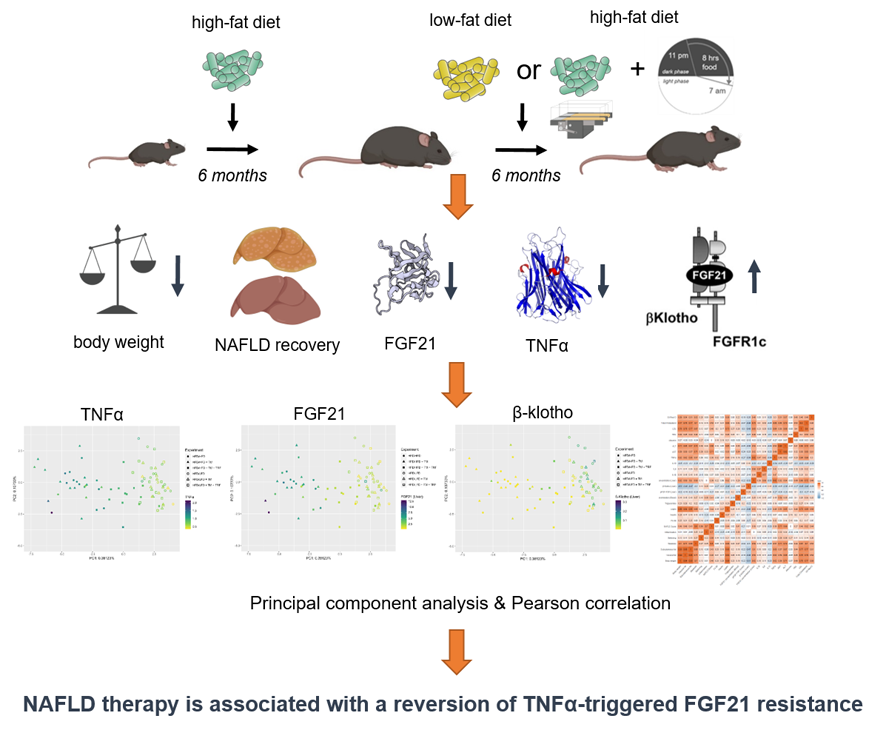

Supplement: Supplementary file 3 [file Image_1.TIF]
